# A gender and size specific evaluation of Grammont-type inlay versus lateralizing onlay stem designs in achieving lateralization and distalization in reverse shoulder arthroplasty

**DOI:** 10.1186/s12891-024-07818-y

**Published:** 2024-09-04

**Authors:** Jan-Phillip Imiolczyk, Paula-Nevin Abu Zeid, Larissa Eckl, Tankred Imiolczyk, Frank Gohlke

**Affiliations:** 1 Center for Musculoskeletal Surgery, Charité Universitaetsmedizin, Berlin, Germany; 2 Department of Shoulder and Elbow Surgery, Schulthess Clinic, Zurich, Switzerland; 3 Department of Mathematics, University of Mannheim, 68131 Mannheim, Germany; 4 Department for Shoulder and Elbow Surgery, Rhoen Klinik, Bad Neustadt/Saale, Germany

**Keywords:** Reverse shoulder arthroplasty, Lateralization, Distalization, Onlay, Inlay, Humeral stem, Gender medicine, Sex, Pink

## Abstract

**Introduction:**

In reverse shoulder arthroplasty (RSA) new designs enable greater amounts of lateralization to prevent instability and scapular notching and increase range of motion, however, excessive lateralization leads to stress upon the acromion that can result in scapular spine fatigue fractures. Aim of this study was to gender- and size-specifically assess the influence of glenosphere size and different humeral designs on lateralization, distalization, and bony impingement-free range of motion (ROM) in patients undergoing RSA.

**Methods:**

Computed tomography scans from 30 osteoarthritic patients (f:15, m:15) and 20 cuff tear arthropathy patients (f:10, m:10) were used to virtually simulate RSA implantation. The efficacy of an inlay Grammont-type system vs. an onlay lateralizing system combined with different glenosphere sizes (36 mm vs. 42 mm) in achieving ROM, lateralization, and distalization was evaluated. Moreover, gender and patient’s constitution were correlated to humeral size by radiologically measuring the best-fit circle of the humeral head.

**Results:**

A different amount of relative lateralization was achieved in both genders using large glenospheres and onlay designs. Latter yielded a higher ROM in all planes for men and women with a 42 mm glenosphere; with the 36 mm glenosphere, an increased ROM was observed only in men. The 155° inlay design led to joint medialization only in men, whereas all designs led to lateralization in women. When adjusting the absolute amount of lateralization to humerus’ size (or patient’s height), regardless of implant type, women received greater relative lateralization using 36 mm glenosphere (inlay: 1%; onlay 12%) than men with 42 mm glenosphere (inlay: -3%; onlay: 8%).

**Conclusion:**

The relative lateralization achieved using onlay design is much higher in women than men. Small glenospheres yield greater relative lateralization in women compared to large glenospheres in men. Humeral lateralization using onlay designs should be used cautiously in women, as they lead to great relative lateralization increasing stress onto the acromion.

**Level of evidence:**

Basic Science Study, Computer Modeling.

**Supplementary Information:**

The online version contains supplementary material available at 10.1186/s12891-024-07818-y.

## Introduction

Reverse shoulder arthroplasty (RSA) is a reliable treatment option to restore joint mobility and reduce pain for cuff-deficient or severe arthritic shoulders. Yet, worse postoperative outcomes have been reported in female patients compared with men [1, 2]. For example, in a cohort of 660 patients undergoing RSA, Friedman et al., reported a better postoperative range of motion (ROM) in male patients, who displayed higher abduction (ABD), forward flexion, and passive external rotation (ER) compared with their female counterparts [1]. This gender disparity can be partially attributed to the anatomical shoulder differences observed between men and women, which influence joint biomechanics. RSA “reverses” the native articular concavities of the glenoid and humerus, which results in several biomechanical advantages relative to the native shoulder [3, 4]. By the medialization of the center of rotation (COR) [4], the traditional “Grammont-designed RSA” improves ROM and reduces the risk of implant failure. However, this technique is not exempt of complications: a scapular notching rate of approximately 75% has been previously described in patients undergoing Grammont-designed RSA, increasing up to 95% among young patients [5]. Lateralization can be imposed by design changes either on the humerus or the glenoid to prevent scapular notching and enhance ROM [6–8]. Lateralization improves the torque generated by remnants of the rotator cuff but decreases the lever arm especially of the mid deltoid muscle and therefore enhances fiber recruitment, which results in greater shearing forces [9, 10]. This augmented load on the acromion can result in an increased incidence of scapular spine fatigue fractures [11]. Glenoid sided lateralization shifts the center of rotation more laterally by lengthening artificially the scapular neck, hence reducing scapular notching, improving stability and shoulder contour as well as the lever arm for the remaining rotator cuff. Humerus sided lateralization merely shifts the greater tuberosity more laterally, increasing deltoid and soft tissue wrapping, increasing stability and improving the natural shoulder contour. Design changes, such as curvature and onlay systems as well as decreasing humeral neck-shaft angle (NSA) are examples of humeral lateralization increasing bony impingement-free ROM, hence reducing scapular notching [8, 12]. Excess lateralization often leads to difficult intraoperative reposition, whereas “overstuffing” can result in pain or poor function. In a clinical comparison the modified onlay system presented superior results in achieving higher ROM and reducing risk of scapular notching than the Grammont design, but these benefits come at the expense of a three-fold increased scapular spine fracture incidence [9, 11]. Notably, those fatigue fractures occur predominantly in women, and a positive correlation with osteoporosis has been established [13, 14]. However, design changes propagating greater levels of lateralization may achieve an even greater impact relatively to size in women due to their smaller size on average. A recent multicentric study shows, that female sex and osteoporosis are both risk factors associated with acromial and spine fatigue fractures [15]. Besides bone mineral density, the use of lateralizing designs bears a higher risk of experiencing fatigue fractures for women [12, 16]. As osteoporosis weakens the bone, it reduces the mechanical breaking strength of the acromion and scapular spine making even smallest changes in design, increasing shearing forces, become really evident. Therefore, we used a virtual computer simulation to evaluate the influence of a lateralizing curved, onlay design with 145° NSA compared to a traditional Grammont inlay 155° stem in addition to glenosphere size on lateralization, distalization, and bony impingement-free ROM with regards to gender and patient’s constitution. We hypothesized that small women will receive relatively greater lateralization with modular onlay systems than men. Our second hypothesis was that inlay and onlay designs yield different ROM. Our null hypothesis was that both stems achieve the same lateralization and ROM in both female and male patients.

## Methods

**Fig. 1:**
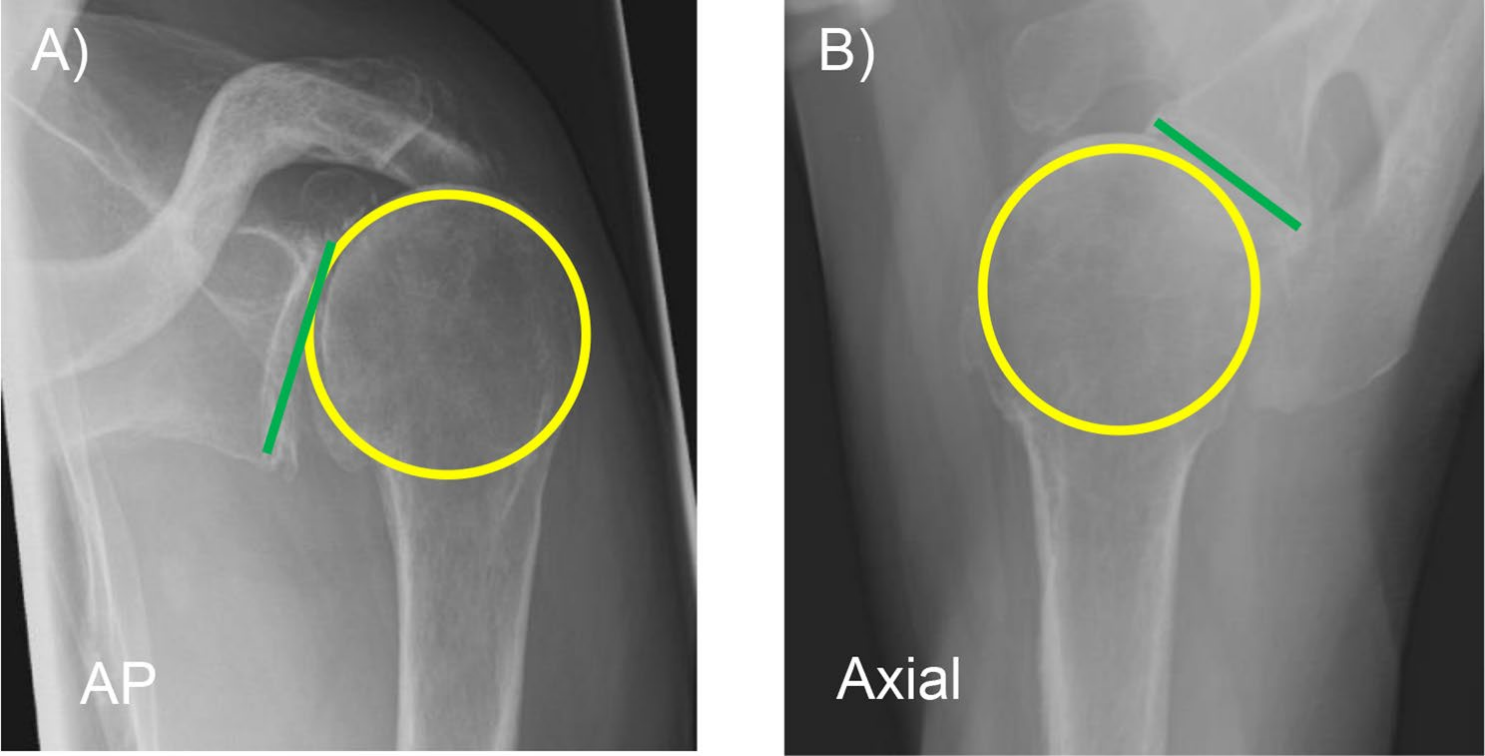
Radiologically determination of best-fit circle of the humeral head in yellow. The humerus height and width appears colored in green in anteroposterior (AP) (**A**) and axial (**B**) radiographs

We analyzed computed tomography (CT) scans and standardized preoperative true anteroposterior (AP) and axial radiographs from 25 male and 25 female patients that have suffered from primary osteoarthritis (OA) (*n* = 30) or cuff tear arthropathy (CTA) (*n* = 20) and were treated with a RSA by the senior author (F.G.). In addition to skeletal size and weight from the patients’ files, radiographic measurements from preoperative x-rays were used to evaluate patients’ constitution more accurately. To determine humeral head size, a best-fit circle was drawn onto the humeral head and aligned from the humeral joint surface up to the greater tuberosity in true AP and axial radiographs, which enabled us to determine best-fit circle’s radius. Glenoid’s height was measured in true AP and glenoid’s width was determined using axial radiographs (Fig. 1). A correlation among these four measurements and patients’ height, weight, and body mass index (BMI) was performed (Tables 1 and 2).

**Table 1:** Patient’s constitution, 2-Dimensional and 3-Dimensional radiographic measurements, and glenoid pathology in female and male patients. For each parameter, the mean value ± standard deviation is represented

	Men	Women
**Patient’s constitution**		
Height (cm)	175.8 ± 6.2 (164–190)	162.6 ± 5.3 (150–172)
Weight (kg)	92.2 ± 16.2 (60–124)	80.4 ± 16.1 (53–130)
BMI (kg/m^2^)	29.9 ± 5.2 (22.2–39.4)	30.4 ± 5.4 (20.4–45.0)
**Radiographic measurements**		
Glenoid height (AP) (mm)	40.2 ± 3.3 (35–48)	34.5 ± 3.3 (29–41)
Glenoid width (axial) (mm)	32.05 ± 5.1 (24–47)	28.0 ± 2.8 (23–35)
Humerus size (AP) (mm)	51.8 ± 3.0 (44–57)	45.2 ± 2.7 (41–54)
Humerus size (axial) (mm)	52.9 ± 2.6 (45–57)	45.7 ± 3.3 (34–51)
**Glenoid pathology**		
Glenoid inclination (°)	8 ± 7 (0–28)	10 ± 6 (1–27)
Glenoid retroversion (°)	12 ± 9 (0–31)	15 ± 9 (0–37)
Posterior humerus subluxation (%)	69 ± 12 (47–89)	74 ± 14 (48–91)

**Table 2:** Correlations between patient’s physical constitution (size, weight, and body mass index (BMI)) and humeral and glenoid anatomy. The humeral radius corresponds to the radius of the best-fit circle drawn in patient’s humeral head as measured in anteroposterior (AP) and axial radiographs

	Humerus radius (AP)	Humerus radius (axial)	Glenoid height (AP)	Glenoid width (axial)
Size correlation	0.80	0.77	0.66	0.45
Weight correlation	0.47	0.49	0.49	0.14
BMI correlation	0.07	0.12	0.19	-0.007

.

All CT scans were processed by a validated three-dimensional (3D) software program (*Glenosys*, Imascap, Brest, France) [17]. This software enables the virtual implantation of the humeral and glenoid components and generates a 3D reconstruction of the joint while measuring glenoid version, inclination, and posterior humerus subluxation by a multiple spot analysis of the scapula [18]. Lateralization and distalization are measured using an automated software algorithm along a scapular plane that is based on all 3D points of the scapular body. Those measurements of lateralization and distalization describe the projected distance of lateral and inferior displacement in position between the initial and planned humerus as vector in 3D space.

**Fig. 2:**
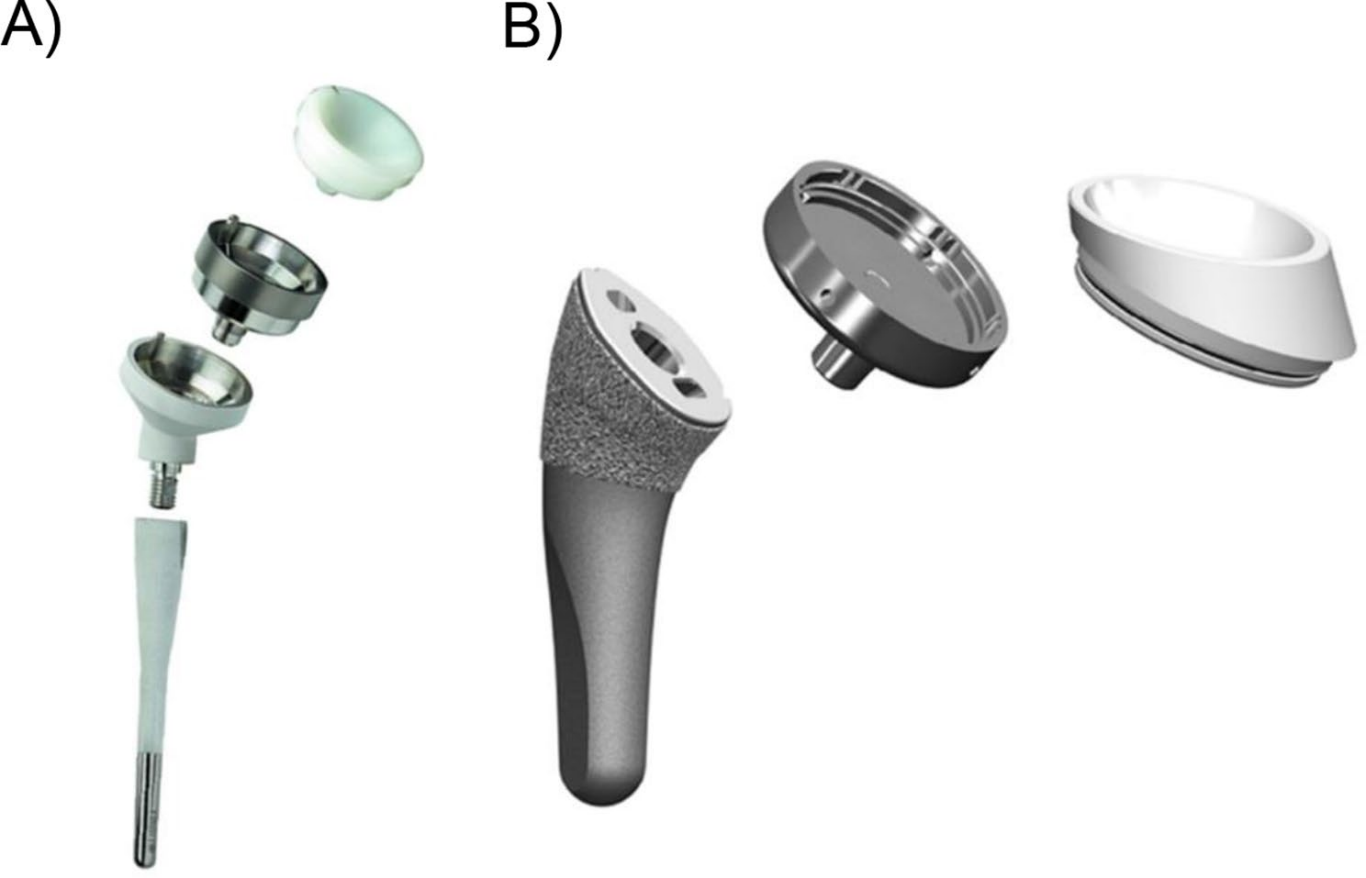
Representation of all the humeral components of the Inlay design Grammont stem “Aequalis Reverse II” (**A**) and the onlay curved stem “Ascend Flex” (**B**). Both implant designs are from Stryker Corp. (Kalamazoo, MI, USA)

On the glenoid side, a standardized 12.5° angled bone block with a maximum height of 13 mm was used for augmentation behind the base plate to neutralize all posterior and cranial glenoid defects similar to those conducted by the surgeon in real life. Depending on the glenoid defect, the width was chosen as this as possible to restoring the native joint line and do not achieve additional glenoid lateralization. This angled bony augmentation was placed to achieve a neutral inclination and version on the inferior glenoid to allow for baseplate placement with no inferior bony overhang at the most lateral point allowing for maximum glenoid seating by the software. In all patients, either a small centered 36 mm glenosphere or a large 42 mm glenosphere with no eccentricity was virtually implanted. On the humeral side two different systems were used: the lateralizing onlay design modular platform Tornier Flex prosthesis (Stryker Corp., Kalamazoo, MI, USA) with a NSA of 145° and the inlay design “Grammont-style” Aequalis Reverse II (Stryker Corp., Kalamazoo, MI, USA) with a NSA of 155° (both shown in Fig. 2). The humeral cut was performed at the anatomic neck with the lateral metaphyseal component aligned at the greater tuberosity.

A RSA was virtually simulated in all 50 patients to reconstruct each patient’s shoulder using four possible configurations: (i) 36 mm glenosphere and 155° inlay stem; (ii) 36 mm glenosphere and 145° onlay stem; (iii) 42 mm glenosphere and 155° inlay stem; and (iv) 42 mm glenosphere and 145° onlay design. Hereafter, the curved 145° design will be referred to as “onlay”, whereas the 155° Grammont-style stem is referred to as “inlay”.

For each configuration, the global ROM defined as bony impingement-free in adduction (ADD), abduction (ABD), forward flexion (FLX), extension (EXT), external rotation (ER) and internal rotation (IR) was tested with the arm at the side. The impingement-free ROM simulation is based on either the collision between two bony structures or the implant and the bone (e.g. scapula or acromion). The software allows for a resolution of 1°. The maximum values for each bony-free impingement ROM were documented for each configuration and patient. Due to the sample size, we have performed (non)-parametric tests. The Man-Whitney-U test was performed for both concurrent factors (i.e., glenosphere size and stem design). Significance level was set at 0.05.

## Results

### Patients characteristics

In our study population, women had a smaller mean (SD) height (162.6 ± 5.3 cm) and lower mean weight (80.4 ± 16.1 kg) than men (175.8 ± 6.2 cm and 92.2 ± 16.2 kg), respectively (Table 1). Accordingly, all two-dimensional (2D) radiographic measurements (i.e. humerus radius, glenoid height, and glenoid width) were smaller in women than in men and are displayed in Table 1. We observed that the greatest correlation between patient’s skeletal size and joint anatomy was defined by the radius of best-fit circus of the humeral head and was therefore used from here on when referring to humeral size (Table 2).

At the pathology level, glenoid inclination and retroversion were on average marginally higher in female than in male patients (Table 1). Patients with primary OA showed on average a greater amount of glenoid retroversion and posterior humerus subluxation compared with CTA patients. A higher glenoid inclination was observed in CTA patients compared to OA patients (Supplementary Table 1).

### Influence of glenosphere size on lateralization and range of motion

**Fig. 3:**
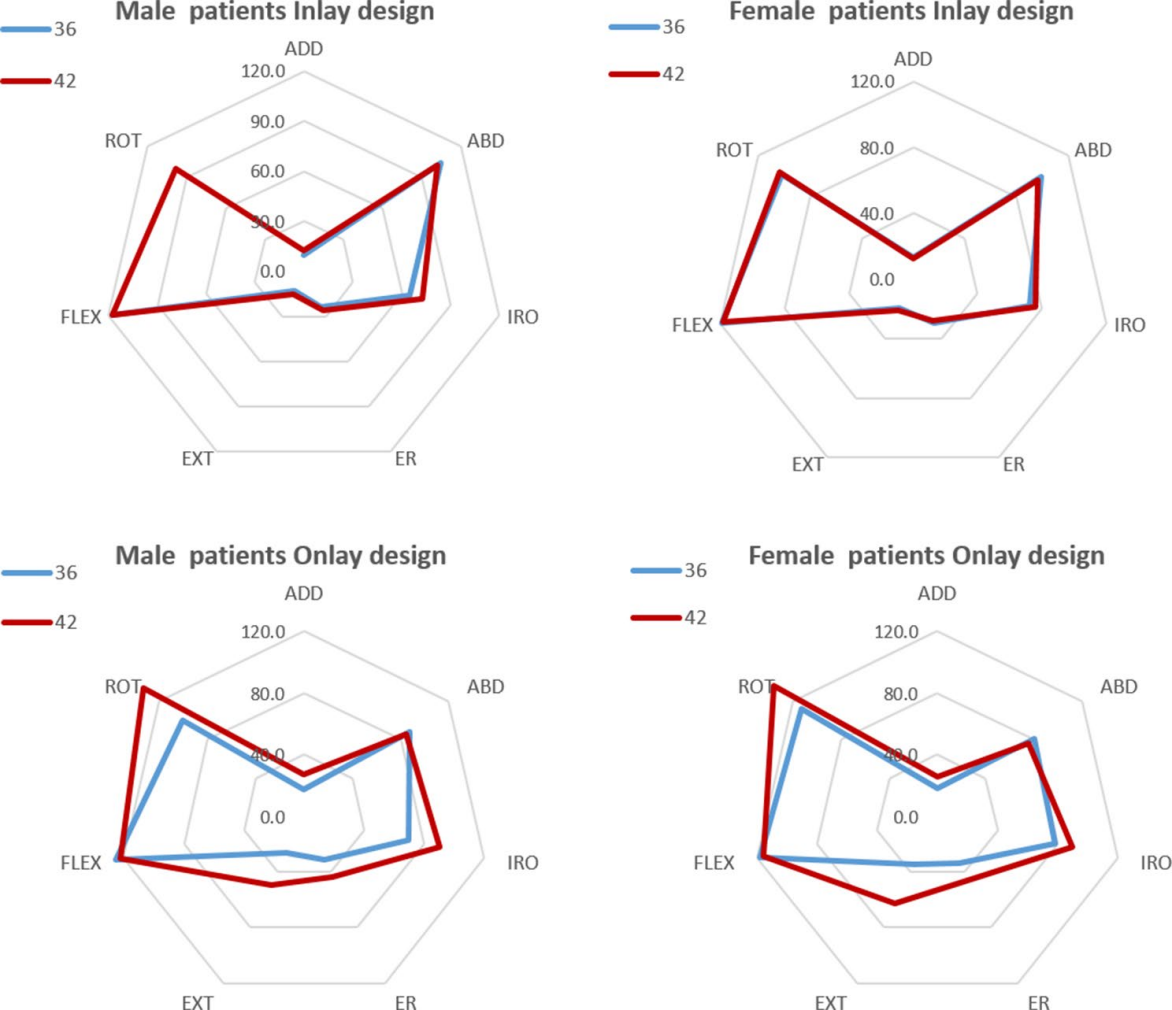
Impact of glenosphere size on range of motion in female and male patients when combined with either inlay designs or onlay designs. ADD: adduction angle (°); ABD: adduction angle (°); IRO: internal rotation angle (°); ER: external rotation angle (°); EXT: extension angle (°); FLEX: forward flexion angle (°); ROT: impingement-free range of motion

The use of a 42 mm glenosphere combined with the onlay system led to greater lateralization in both genders compared to the one achieved when using a 36 mm glenosphere (Table 3). The results for pairwise comparison achieved with each configuration are presented in Table 3; Fig. 3. A significant increase in ADD (*P* ≤ 0.046), EXT (*P* ≤ 0.018), and ER (*P* ≤ 0.025) was observed in both genders with the use of a 42 mm glenosphere and an onlay implant, which depicts a greater impingement free zone with the arm at the side. In contrast, an increased glenosphere size in combination with the inlay design did not improve ROM either in men nor in women, with the exception of IRO in men (Table 3; Fig. 3). Forward flexion was not improved when increasing glenosphere size in any of the cohorts regardless of the implant type.

**Table 3:** Pairwise comparison of the influence of glenosphere size on range of motion for all four configurations. All parameters are reported in the mean value

Variable	men			women			men			women		
	inlay			inlay			onlay			onlay		
	36	42	*P*	36	42	*P*	36	42	*P*	36	42	*P*
Lateralization (mm)	-3.0	-1.9	0.450	0.6	1.6	0.288	1.3	4.1	0.016	5.4	8.6	0.001
Distalization (mm)	-34.5	-34.4	0.973	-28.6	-28.5	0.975	1.3	4.1	0.016	-27.4	-27.9	0.725
Adduction (°)	9.8	12.4	0.264	13.5	12.7	0.753	17.6	26.9	0.002	18.6	25.6	0.046
Abduction (°)	104.5	102.1	0.340	99.4	96.5	0.444	87.9	85.2	0.341	80.9	76.0	0.197
Int. Rotation (°)	64.8	72.8	0.049	72.6	76.0	0.569	69.3	89.9	0.001	78.7	89.8	0.080
Ext. Rotation (°)	24.0	25.7	0.704	29.2	28.0	0.816	31.2	43.2	0.005	33.6	45.8	0.025
Extension (°)	13.3	15.2	0.552	19.6	21.0	0.827	26.1	49.1	0.009	34.8	63.0	0.018
Forward Flexion (°)	117.9	117.9	1.000	118.6	118.0	0.921	125.5	122.5	0.647	118.4	115.4	0.597
Rot. Range (°)	88.8	98.5	0.103	101.8	104.0	0.819	100	133	0.001	112	136	0.022

### Influence of stem design on lateralization, distalization, and range of motion

**Fig. 4:**
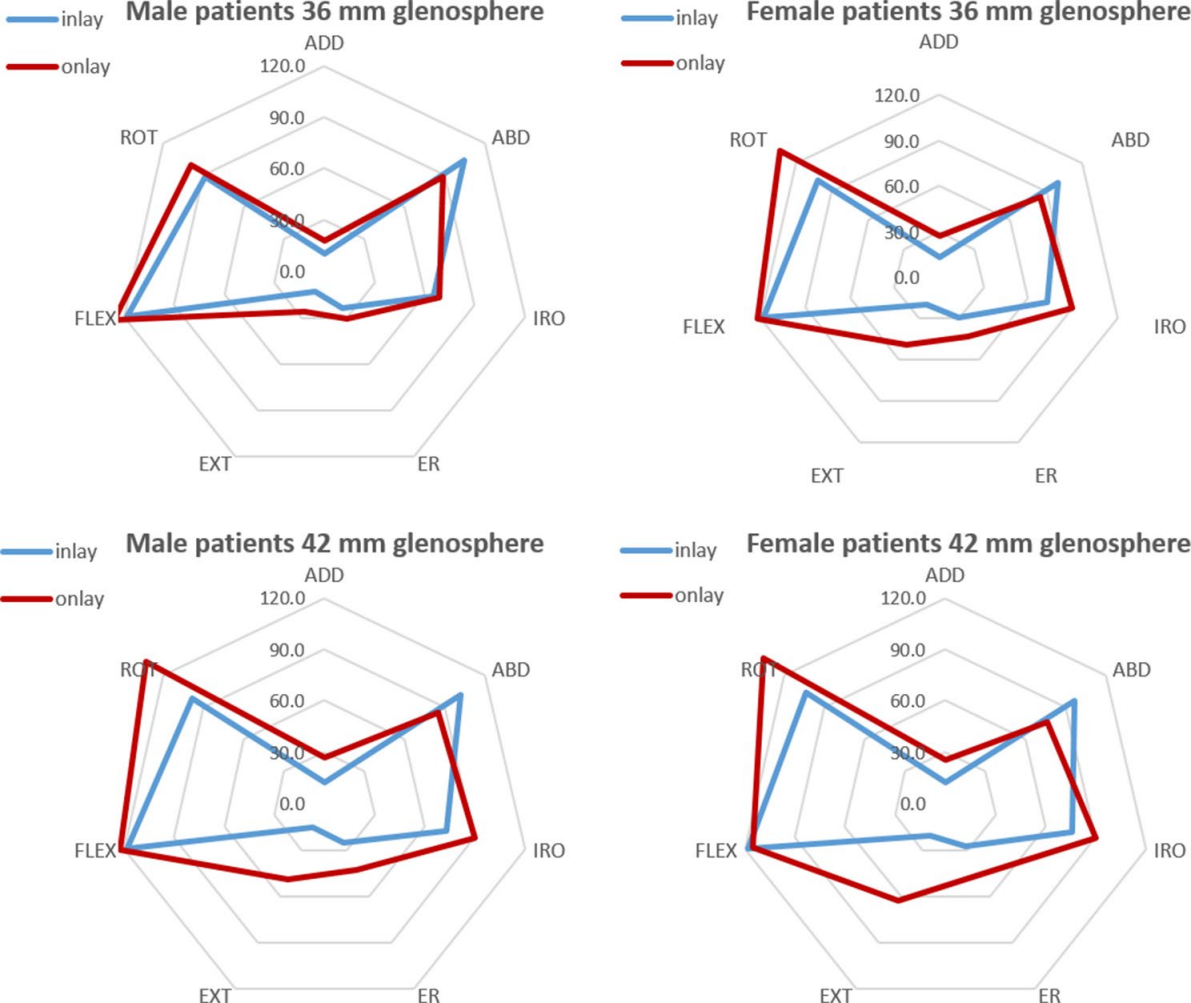
Impact of humeral component design on range of motion in female and male patients when combined with either a 36 mm glenosphere or a 42 mm glenosphere. ADD: adduction angle (°); ABD: adduction angle (°); IRO: internal rotation angle (°); ER: external rotation angle (°); EXT: extension angle (°); FLEX: forward flexion angle (°); ROT: impingement-free range of motion. All parameters reported represent the mean value of each category

Onlay systems were superior over inlay systems in achieving a higher level of lateralization, regardless of the glenosphere size or gender (Table 4). The pairwise comparison for each parameter and each configuration is shown in Table 4. When combined with a 42 mm glenosphere, the onlay system yielded a significant improvement in both men and women in ADD (*P* ≤ 0.001), ABD (*P* ≤ 0.001), IRO (*P* ≤ 0.023), ER (*P* ≤ 0.001), and EXT (*P* ≤ 0.001) (Fig. 4). However, no significant differences were observed in the amount of distalization achieved by both implants. The inlay design, however, led to an impingement between greater tuberosity and the acromion, which increased abduction in this static shoulder simulation. No significant changes in distalization were observed with both type of implants (Table 4).

**Table 4:** Pairwise comparison of the impact of onlay and inlay design in combination with either a 36 mm or a 42 mm glenosphere on range of motion. All parameters reported represent the mean value of each category

Variable	men			women			men			women		
	36 mm			36 mm			42 mm			42 mm		
	inlay	onlay	*P*	inlay	onlay	*P*	inlay	onlay	*P*	inlay	onlay	*P*
Lateralization (mm)	-3.0	1.28	0.002	0.6	5.4	0.001	-1.9	4.08	0.001	1.6	8.60	0.001
Distalization (mm)	-34.5	-33.80	0.608	-28.6	-27.4	0.380	-34.4	-34.88	0.752	-28.5	-28	0.630
Adduction (°)	10.0	18	0.002	13.5	18.6	0.087	12.4	27	0.001	12.7	26	0.001
Abduction (°)	104.0	88	0.001	99.4	80.9	0.001	102.1	85	0.001	96.5	76	0.001
Int. Rotation (°)	65.0	69	0.427	72.6	78.7	0.338	72.8	90	0.001	76.0	90	0.023
Ext. Rotation (°)	24.0	31	0.127	29.2	33.6	0.390	25.7	43	0.001	28.0	46	0.001
Extension (°)	13.0	26	0.014	19.6	34.8	0.066	15.2	49	0.001	21.0	63	0.001
Forward Flexion (°)	118.0	126	0.316	118.6	118.4	0.965	117.9	122	0.497	118.0	115.44	0.676
Rot. Range (°)	89.0	100	0.076	101.76	112	0.277	98.5	133	0.001	104	136	0.003

### Absolute and relative lateralization depending on patient’s constitution

**Fig. 5:**
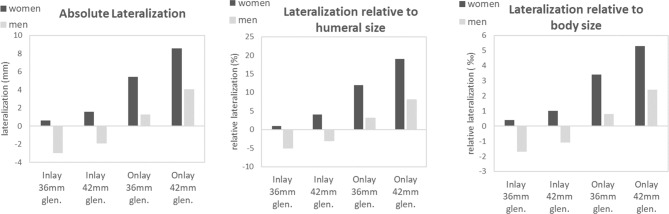
Absolute and relative lateralization achieved by each configuration. All parameters reported represent the mean value of each category. Lateralization relative to humeral head size was calculated using the radius of best-fit circle and lateralization relative to body size was calculated using patient’s height

Comparison of the absolute and relative lateralization achieved by each configuration is displayed in Fig. 5. The greatest amount of lateralization in male patients was obtained with the use of onlay systems combined with a 42 mm glenosphere, which resulted in an absolute lateralization of 4.08 mm and represents an increase of 8% of lateralization relative to the humeral size. However, a similar degree of absolute lateralization (5.44 mm) can be accomplished in female patients with the use of smaller glenospheres, which corresponds to a relative lateralization of 12% (Fig. 5). Indeed, onlay system combined with a 42 mm glenosphere yielded an increase of 19% relative lateralization in women, which is almost three times the amount of lateralization achieved in men with the optimal configuration (Fig. 5).

## Discussion

RSA designs are evolving towards systems that increase lateralization both on the glenoid and humeral side, with the ultimate goal to prevent scapular notching and improve ROM [19]. However, the “ideal amount of lateralization”, especially in female patients, remains unknown to date. The purpose of our study was to evaluate the biomechanical effects of glenoid and humeral lateralization on ROM on female and male patients. As variations in implant design impact on shoulder kinematics, and in turn, on functional outcomes, we performed a gender-specific virtual simulation of a RSA with onlay and inlay systems combined with the two common glenosphere sizes.

Our study showed that increasing humeral lateralization with the use of onlay systems had a greater effect in achieving free-impingement passive ROM compared with the use of larger glenospheres. Even when combined with 36 mm glenospheres, the use of onlay systems in women, whose physical constitution tends to be smaller than men, resulted in a relative lateralization much higher than in men implanted with large glenospheres. This may explain the three-fold increase in fatigue fractures when using onlay design stems [12]. Our data suggests that women are more susceptible to greater amounts of lateralization compared with men by design. There is a higher degree of relative lateralization observed in female patients regardless of implant design.

We observed that the use of large glenospheres translated into higher lateralization and greater impingement-free ROM in silico. Moreover, the use of smaller glenosphere in women yielded in a greater relative lateralization compared to those for men using the larger glenosphere. These results are in agreement with previous studies, where glenoid lateralization can improve ROM regardless of the humeral inclination [8, 20, 21]. In a cadaveric study, Berhouet et al., demonstrated that greater ADD and free-impingement ROM was achieved when using a 42 mm glenosphere than when using a 36 mm glenosphere [21]. A higher level of lateralization was theorized to minimize scapular notching. This was recently demonstrated in a multicenter clinical study including 61 female and 41 male patients undergoing RSA and receiving either a medialized or lateralized glenosphere. While the reported scapular notching rate was lower in the lateralized group, the incidence of scapular spine fractures was similar between groups [22].

Our study demonstrated that humeral lateralization together with 145° inclination had the greatest impact on impingement-free passive ROM compared to the classic Grammont-design. Comparison of ROM achieved by inlay versus onlay designs revealed superior results in ADD, EXT, and ER angles for both genders with the use of onlay systems. The use of inlay systems improved IRO, but this improvement was only significant for men. Our results supports Werthel’s theoretical findings that more than double of lateralization can be achieved on the humeral side compared with the glenoid side [23]. However, we found that ABD was significantly lower when increasing humeral lateralization, probably due to the lateralization of the greater tuberosity and abutment at the acromion which in vivo may be compensated by scapulothoracic movement, which is not present in our simulating system.

Contrary to lateralization, which clearly increased with the use of onlay systems, no significant changes in distalization were observed with the use of any type of implant. On the one hand, different resection angles used using a 145° and 155° NSA when the lateral metaphyseal component is aligned to the greater tuberosity. This results in a more proximal cut at the medial calcar and relatively less distalization. On the other hand, onlay systems do increase distalization. This combination may explain the lack of differences in distalization when using different stem systems. The increase in glenosphere size results in a mean increase of distalization of 1 mm regardless of implant configuration.

We postulate that with the use of implants currently available on the market, and the male patients-based assumption that a higher lateralization improves ROM, women are predisposed to receive an excessive amount of lateralization relative to their joint and body sizes. While the classical 155° Grammont inlay prosthesis led in our study to the medialization of the COR in men, the same design already led to lateralization of COR and the onlay design resulted in a high degree of relative lateralization in female patients. Indeed, we observed that women with a 36 mm glenosphere and onlay design obtained over 4% of lateralization more (compared to joint size) than men when using a 42 mm glenosphere and onlay design. These totally disproportionate effects of standard implant sizes on men and women should be targeted by prostheses’ manufactures, as there should be a greater variety of component sizes on the market to allow for individual risk analysis.

Limitations of this study include that the evaluation of implants was restricted to one platform onlay design versus one curved inlay stem design, and that both were from the same manufacturer. Moreover, both stems had different NSA, which has been reported to influence the lateralization achieved on the humeral side [8]. Taken together, our results cannot be extrapolated to other prostheses available in the market, as a more comprehensive and precise comparison needs to be performed to reach conclusion about the optimal implant design for each gender [8, 24, 25]. An additional limitation is that a mixed configuration (i.e. 42 mm glenosphere with a 36 mm stem) could not be simulated with our software, even if such configuration is often used by surgeons and represents a viable option for some patients. This configuration presents several advantages over the ones above-described, such as greater ADD range with less lateralization. Furthermore, the computational simulation did not enable us to evaluate neither scapulothoracic movement nor soft tissue tensioning with regards to pre-tensioning of the delta muscle or the remaining cuff. Strength of this study is, that those two implant configurations represent the traditional Grammont design (155° inlay design) with one of the most common designs allowing for humeral lateralization.

Our study suggests that surgeons should evaluate lateralization individually for each patient by 3D software-based planning and thoroughly assess the trade-off between increased ROM and biomechanical side effects based on patient’s constitution, gender, and bone quality.

## Conclusion

In reverse shoulder arthroplasty, relative lateralization is much higher in women, especially when using an onlay designs and large glenospheres. Humeral lateralization using new modular onlay systems should be used cautiously especially for small women. Small glenospheres yield greater relative lateralization in women compared to large glenospheres in men. Glenoid lateralization can help to reduce impingement by increasing ROM and can therefore be used more effectively for small women.

## Electronic supplementary material

Below is the link to the electronic supplementary material.

Supplementary Material 1

## Data Availability

The datasets used and/or analyzed during the current study available from the corresponding author upon reasonable request.
