# Supplementary material for: A gender and size specific evaluation of Grammont-type inlay versus lateralizing onlay stem designs in achieving lateralization and distalization in reverse shoulder arthroplasty
Source: BMC Musculoskelet Disord. 2024 Sep 4;25:709. doi: 10.1186/s12891-024-07818-y (PMC11373514; doi:10.1186/s12891-024-07818-y)
Supplement: Supplementary file 1 — Supplementary Material 1 [file 12891_2024_7818_MOESM1_ESM.docx]

**Supplementary Table 1**

|  | OA  (n=30)  Mean (SD)  range | CTA  (n=20)  Mean (SD)  range | total  (n=50)  Mean (SD)  range |
| --- | --- | --- | --- |
| Glenoid inclination (°) | 8 (7)  (0-27) | 10 (7)  (1-28) | 9 (7)  (0-28) |
| Glenoid retroversion (°) | 17 (9)  (0-37) | 9 (8)  (0-26) | 13 (8)  (0-37) |
| Posterior humerus subluxation (%) | 77 (11)  (48-91) | 63 (12)  (47-91) | 71 (12)  (47-91) |
| Glenoid erosion according to Walch^23, 24^ or Favard ^25^ | 1 x A1  5 x A2  3 x B1  10 x B2  7 x B3  4 x C | 3 x E0  9 x E1  4 x E2  4 x E3 |  |

**Supplementary Table 1**: Study population investigated with regards to 3D measured glenoid inclination, version and, humerus subluxation for all patients and both diagnoses separately
